# Titanium Particles Modulate Lymphocyte and Macrophage Polarization in Peri-Implant Gingival Tissues

**DOI:** 10.3390/ijms241411644

**Published:** 2023-07-19

**Authors:** Waad Kheder, Amal Bouzid, Thenmozhi Venkatachalam, Iman M. Talaat, Noha Mousaad Elemam, Tom Kalathil Raju, Soumya Sheela, Manju Nidagodu Jayakumar, Azzam A. Maghazachi, Abdul Rani Samsudin, Rifat Hamoudi

**Affiliations:** 1 College of Dental Medicine, University of Sharjah, Sharjah 27272, United Arab Emirates; 2 Research Institute for Medical and Health Sciences, University of Sharjah, Sharjah 27272, United Arab Emirates; 3 College of Medicine, University of Sharjah, Sharjah 27272, United Arab Emirates; 4 Division of Surgery and Interventional Science, University College London, London NW3 2PS, UK; 5 ASPIRE Precision Medicine Research Institute Abu Dhabi, University of Sharjah, Sharjah 27272, United Arab Emirates

**Keywords:** dental implants, peri-implantitis, titanium, lymphocytes, macrophage polarization, IL-1β, IL-18, IL-8

## Abstract

Titanium dental implants are one of the modalities to replace missing teeth. The release of titanium particles from the implant’s surface may modulate the immune cells, resulting in implant failure. However, little is known about the immune microenvironment that plays a role in peri-implant inflammation as a consequence of titanium particles. In this study, the peri-implant gingival tissues were collected from patients with failed implants, successful implants and no implants, and then a whole transcriptome analysis was performed. The gene set enrichment analysis confirmed that macrophage M1/M2 polarization and lymphocyte proliferation were differentially expressed between the study groups. The functional clustering and pathway analysis of the differentially expressed genes between the failed implants and successful implants versus no implants revealed that the immune response pathways were the most common in both comparisons, implying the critical role of infiltrating immune cells in the peri-implant tissues. The H&E and IHC staining confirmed the presence of titanium particles and immune cells in the tissue samples, with an increase in the infiltration of lymphocytes and macrophages in the failed implant samples. The in vitro validation showed a significant increase in the level of IL-1β, IL-8 and IL-18 expression by macrophages. Our findings showed evidence that titanium particles modulate lymphocyte and macrophage polarization in peri-implant gingival tissues, which can help in the understanding of the imbalance in osteoblast–osteoclast activity and failure of dental implant osseointegration.

## 1. Introduction

The use of dental implants to replace missing teeth is beneficial and increasing due to the reliable and durable performance of implant-supported prostheses. This modality to replace missing teeth is expected to continue in the future as a result of the global population aging and advancements in dental treatments. Though dental implants display high success rates, it is important to note that the challenges in this approach of treatment are the failure of approximately 5% to 11% of the implants within 10 to 15 years and the necessity of removal of the implants [1]. The primary causes of this failure are attributed to biological or biomechanical factors; these factors are complex and interrelated. However, peri-implantitis is an inflammatory process that affects peri-implant tissues, leading to the loss of supporting bone. Therefore, clinical interventions aim to preserve the integrity of peri-implant soft and hard tissues and increase the success of dental implant treatment [2].

Titanium is the metal of choice for oral and maxillofacial implants. It is a highly reactive metal that develops a passivating layer of titanium dioxide (TiO_2_) over its surface upon exposure to air or fluids. This TiO_2_ layer acts as a crucial interface between the implant and the biological milieu, ensuring biocompatibility by reducing material reactivity and partially preventing corrosion [3]. However, the loss of this protective layer without reformation could lead to corrosion similar to the other base metals [4]. The biofilms produce acids as byproducts of their metabolic activities, which can lead to erosion of root structures and deterioration of dental prosthetic materials over time. However, polymer/polymer-based products and dental adhesives are typically designed to release or generate alkaline substances, which neutralize the acids and create a less acidic environment. This will help to counteract the effects of biofilm acids by providing a protective barrier. Moreover, the adhesives have the potential to achieve superior reductions in biofilm growth, early attachment, and metabolic activity [5,6].

Titanium particles may release into the peri-implant gingival tissues as a result of implant placement surgery, corrosion of the implant surface, micro-movements between the implant and its superstructure, and instrumentation of the implant surface during implant maintenance [7]. In the oral environment, titanium faces fluctuating pH values due to the presence of bacterial biofilm, fluorides, and dietary factors. This may induce an acidic medium that in turn activates the dissolution of titanium particles and disruption of the TiO_2_ layer on the dental implant’s surface [8,9]. These particles act as foreign bodies to the immune cells, triggering several immune responses and consequently promoting the activation of different mediators that are associated with bone resorption and peri-implant diseases [10].

A wide variety of immune cells are present in the peri-implant gingival tissues, such as plasma cells, lymphocytes, neutrophils, granulocytes, and/or macrophages [11]. Titanium particles that have been shed from dental implant surfaces have been identified as a predominant foreign body in peri-implantitis tissue biopsies which are surrounded by inflammatory cells [12]. Those particles are engulfed by macrophages which release pro-inflammatory cytokines such as IL-1β and TNF-α. These cytokines are associated with osteoclast activation via the receptor activator of nuclear factor ĸB ligand (RANKL)/receptor activator of nuclear factor ĸB (RANK)/osteoprotegerin (OPG) signaling pathway. This ultimately leads to osteolysis and bone resorption, which are strongly associated with the development and progression of peri-implantitis [13]. Oliveira et al. showed that macrophages on peri-implant tissues phagocytozed titanium microparticles’ corrosion products, which led to the stimulation of the osteoclasts’ activity, and that most of the interaction between cells of the immune system is modulated by cytokines, growth factors, and hormones [14]. Pro- and anti-inflammatory cytokines have been proposed in influencing the progression of peri-implantitis. Imbalances in the levels of cytokine release may inhibit the resolution of inflammation and contribute to alveolar bone loss [15].

Despite the inconsistencies in definitions of peri-implantitis, disease prevalence varies between 1 and 47% of patients [16]. Infiltration of immune cells significantly affects the biocompatibility and function of dental implants, which can lead to their failure [17]. The initial injury of peri-implant tissues following implant placement triggers an inflammatory response mediated by cells of innate immunity, such as macrophages, dendritic cells, mast cells, and neutrophils. Macrophages play a dual role as either inflammatory (M1 macrophages) or alternative (M2 macrophages) [18]. Oliveira and co-workers reported that, in the presence of titanium particles, osteoclast activity was activated and the number of macrophages in the site was increased, associated with a higher rate of mutations in human cells cultured in titanium-based nanoparticles [14]. Moreover, in vitro research showed that the presence of titanium particles resulted in an increase in the expression of inflammatory cytokines, activation of osteoclasts and morphologic alterations, such as neutrophils and macrophages. Degradation products can cause morphologic alterations in inflammatory cells, consequently increasing the release of TNF-α [19,20]. Furthermore, cytokines such as TNF-α and IL-1β were known to induce the expression and activate the promoter of CCL3, which was reported to play a critical role in the potential migration of macrophages [21]. During inflammation, leukocyte recruitment is regulated by various cytokines, including IL-8/CXCL8, CCL2, IL-1β, and TNFα, which act as prominent chemoattractants [22].

However, there is a gap in the knowledge about the role of titanium particles in the development of peri-implantitis and the failure of dental implant osseointegration. Exploring this area of research can shed light on the relationship between titanium particle infiltration into peri-implant tissues and the modulation of the immune cells, thereby enhancing our understanding of implant osseointegration failure. This will contribute to the understanding of the imbalance in osteoblast–osteoclast activity and the failure of dental implant osseointegration. Therefore, the primary objective of this study is to investigate the infiltration of titanium particles and immune cells into the peri-implant gingival tissues by conducting a comparative gene expression analysis using whole transcriptome analysis on gingival tissues surrounding both successful and failed dental implants, in comparison to a reference group without implants. The subsequent step was functional validation using THP-1 monocyte-derived macrophage cell lines to confirm findings and provide additional insights.

## 2. Results

### 2.1. Transcriptional Profiling Distinguishes between FI, SI and NI Gingival Tissue Samples

**Figure 1:**
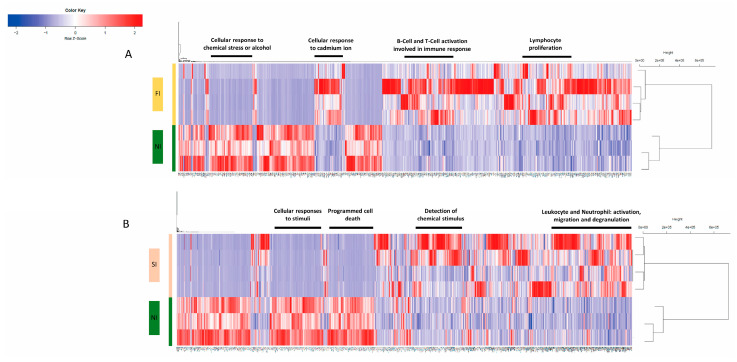
Hierarchical clustering and heatmaps of (rows) the significant DEGs (FDR < 0.05) showing subgroups: (**A**) Failed implants and no implants clusters. (**B**) Successful implants and no implants clusters. The FI, SI, and NI subgroups are indicated by different color schemes. On the heatmaps, red represents upregulated genes and blue represents downregulated genes.

The differential gene expression analysis showed distinct gene expression profiles among FI and SI in comparison to NI gingival tissue samples. After standard normalization and filtering, a total of 2185 and 5375 DEGs resulted in FI compared to NI and SI compared to NI, respectively. The differentially expressed genes were selected with FC > 2 at a significance level of the adjusted *p*-value (FDR) < 0.05 and used for unsupervised hierarchical clustering analysis. The patients with FI and NI are clustered as a single branch, clearly showing subgroups of FI and NI clusters irrespective of their origin from different patients with FI and NI (Figure 1A). In addition, the patients with SI and NI are clustered as a single branch, clearly showing subgroups of SI and NI clusters irrespective of their origin from different patients with SI and NI (Figure 1B). The gene tree in each unsupervised hierarchical clustering was ordered according to the enriched biological pathways by the Metascape annotation tool, and the gene clusters enriched for the top enriched pathways are indicated. Hence, the transcriptomic profiling found in the heatmaps shows a unique signature between FI vs. NI and SI vs. NI, which confirms the immune fraction and their related pathways in each subgroup.

### 2.2. Dysregulation of Cellular Response to Chemical Stress and Immune Response in Gingival Tissues from SI and FI 

To investigate the functional relevance of the significant DEGs in the gingival tissues from FI and SI compared to tissues from NI, functional and pathway analyses were performed (Table 1). The analyses revealed key cellular pathways such as the cellular response to cadmium ion (*p* < 0.01) and lymphocytes B-Cells and T-Cells activation (*p* < 0.0001 and 0.01, respectively) and proliferation processes (*p* < 0.001) that were over-enriched in FI compared to NI. In contrast, the cellular response to chemical stress (*p* < 0.0001) and response to alcohol were significantly downregulated in FI compared to NI. Interestingly, different pathways related to the immune response and process and the activation of immune cells were upregulated in SI, such as leukocyte migration (*p* < 0.0001), myeloid leucocyte activation (*p* < 0.00001), neutrophil and leukocyte degranulation (*p* < 0.0001, 0.001, respectively), in addition to the regulation of cytokine production (*p* < 0.001) and mainly the positive regulation of interleukin-10 production (*p* < 0.001). Moreover, the cellular defense response (*p* < 0.001) and apoptotic cell clearance (*p* < 0.001) pathways were highly enriched in SI compared to NI. However, the functional clustering significantly revealed that cellular responses to stimuli (*p* < 1 × 10^−53^) and Programmed Cell Death (*p* < 1 × 10^−14^) were downregulated in SI compared to NI.

### 2.3. GSEA Identifies the Activated Cellular Pathways in Gingival Tissues from SI and FI 

To identify more detailed pathways and the related DEGs in each comparison of FI vs. NI and SI vs. NI, an exhaustive GSEA was performed. The differentially activated significant pathways were identified for each comparison based on the cut-off nominal *p* < 0.05. The details of the significantly enriched gene sets are listed in Table 2. 

**Figure 2:**
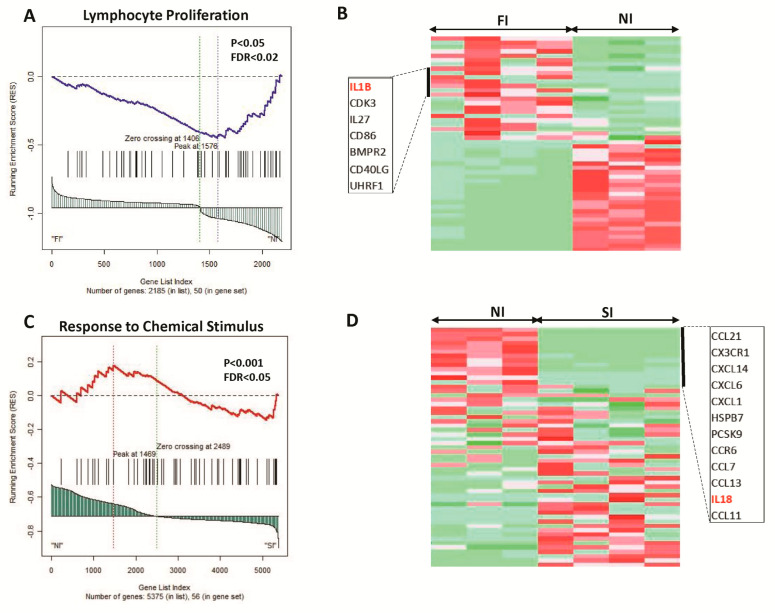
GSEA of (**A**,**B**) Lymphocyte proliferation in FI compared to NI. (**B**,**C**) Response to chemical stimulus in SI compared to NI. (**A**) The distribution of lymphocyte proliferation genes according to their rank position. (**B**) Heatmap illustration of their expression between FI and NI samples. (**C**) The distribution of Response to Chemical Stimulus genes according to their rank position. (**D**) Heatmap illustration of their expression between NI and SI samples. The top leading-edge core genes are shown alongside the heatmaps. FI: Failed implants; SI: Successful implants; NI: No implants.

Further, the leading-edge analyses revealed important differentially activated genes that are involved in multiple inflammations and immune responses related to biological processes or molecular pathways, including IL1B, CDK3, IL27 and CD86 in FI vs. NI comparison (Figure 2A,B), in addition to CXCL6, CXCL1, CCL7, CCL13 and IL18 in SI vs. NI comparison (Figure 2C,D).

### 2.4. Polarization of Macrophage in Peri-Implant Gingival Tissues

**Figure 3:**
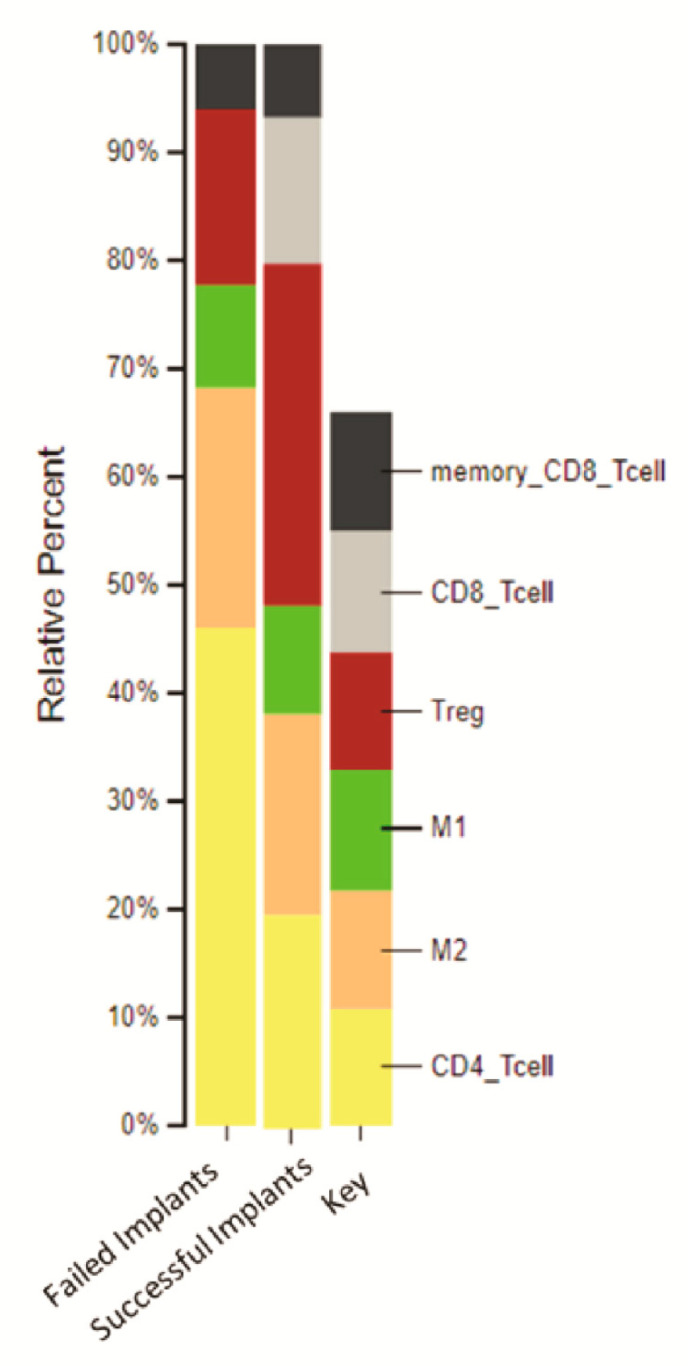
Stacked bar chart format estimating inflammatory cells in the peri-implant gingival tissues of FI and SI compared to NI.

A focused analysis of the deconvolution of immune cells for the DEGs in SI and FI compared to NI was assessed using the CIBERSORT computational method (Figure 3). The digital cytometry analysis showed that the percentage of M1 macrophages was 9.9% in SI, slightly higher than that in FI (9.4%). However, the percentage of M2 macrophage was 22.3% in FI, which was more than that in SI (18.7%). Interestingly, there was a clear variation in the percentages of T cell subtypes between FI and SI samples; for instance, the percentages of regulatory T cells were 16.4% in FI and 3.15% in SI (Table 3). 

### 2.5. Titanium Particles Result in Triggering the Immune System in Both FI and SI

**Figure 4:**
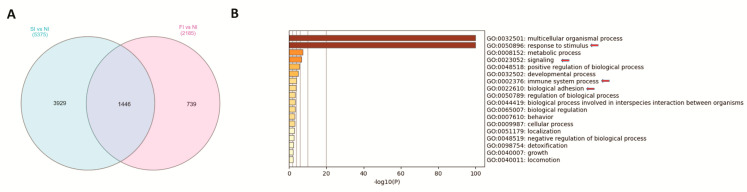
(**A**) Venn diagram representation of the overlap of significantly differentially expressed genes amongst the failed and successful implants. (**B**) Functional clustering and pathway analysis of the common differentially expressed genes across failed and successful implants.

The count of the DEGs across the two types of peri-implant gingival tissues (FI and SI) was investigated (Figure 4A). Functional clustering and pathway analysis of the common DEGs in FI and SI peri-implant gingival tissues showed significant enrichment of transcripts involved in response to stimulus, immune system defense, detoxification and signaling (Figure 4B). 

### 2.6. Higher Infiltration of Immune Cells in Peri-Implant Gingival Tissues of FI Compared to SI or NI

**Figure 5:**
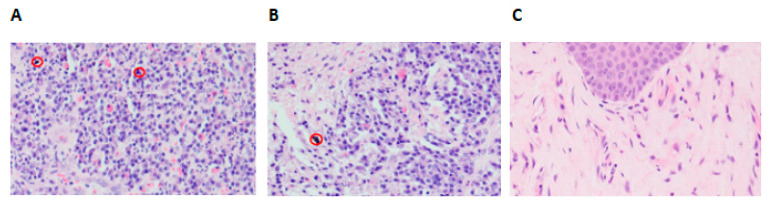
Representative H&E images demonstrating infiltration of inflammatory cells in peri-implant gingival tissues (200×). (**A**) H&E image showing inflammatory cells in the surrounding area of the titanium particles (red circles) in failed implants. (**B**) H&E image showing the titanium particles surrounded by moderate cellular infiltrates in successful implants. (**C**) H&E image showing gingival tissues composed of outer epithelium and inner network of connective tissues in no implants samples.

To confirm immune cell infiltration in peri-implant gingival tissues of FI compared to SI and NI, H&E staining was performed. Microscopic examination showed infiltration of more inflammatory cells into the gingival tissues which were collected from patients with FI, compared to gingival tissues from SI. The predominance of macrophages and lymphocytes is an indication of chronic inflammation. Indeed, the specification of the immune cells was based on the morphology of each cell type based on the nucleus and cytoplasm. Furthermore, titanium particles were evident in the H&E images in the peri-implant gingival tissues collected from patients with FI and SI. However, the gingival tissues collected from patients without dental implants revealed none to minimal infiltration of inflammatory cells (Figure 5). The identification of titanium particles was based on the morphology as well; they are completely different from the inflammatory cells as they look like black spots.

### 2.7. Immunohistochemistry Analysis Validates the Infiltration of Macrophages and Lymphocytes into Peri-Implant Gingival Tissues

**Figure 6:**
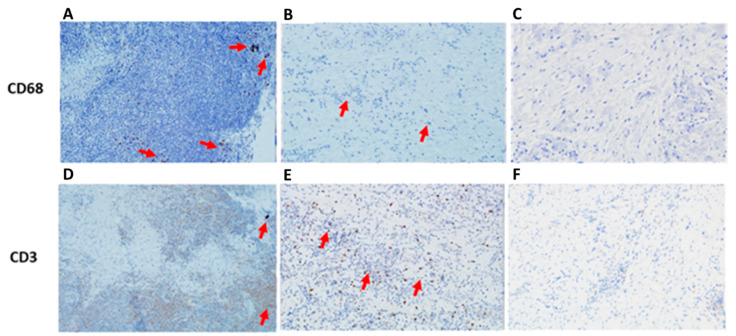
Representative images of CD68 and CD3 IHC staining in peri-implant gingival tissues among the various implant groups (200×). (**A**) IHC staining reveals a strong expression of CD68 in the presence of titanium particles in FI. (**B**) IHC staining reveals CD68 positive expression in the SI but less than that in the FI (**A**). (**C**) IHC staining showed CD68 negative expression in the NI. (**D**) IHC staining reveals CD3 positive expression in the FI. (**E**) IHC staining reveals CD3 positive expression in the SI. However, the proportion of CD3 is more in the FI, and the intensity of staining is strongest in the SI. (**F**) IHC staining showing CD3 negative expression in the gingival tissues of the NI. FI: Failed implants; SI: Successful implants; NI: No implants. The red arrows refer to the expression of the macrophages/ lymphocytes and/or to the titanium particles.

In order to validate the infiltration of immune cells represented by mainly macrophages and lymphocytes in the peri-implant gingival tissues, an immunoreactive score (IRS) using IHC analysis findings was performed to evaluate the expression of CD68 and CD3 markers representative of macrophages and lymphocytes, respectively. Analysis of IHC images showed positive expression of CD68 and CD3 in cases of FI, which was more pronounced than that in SI. In addition, the gingival tissues collected from patients without dental implants showed negative expression of the same markers (Figure 6). Statistical analysis of the immune markers between the various implant groups showed that both CD68 and CD3 were significantly higher in the FI group compared to the SI group (*p* < 0.01), and both were slightly less significant when comparing the NI group to the FI group (*p* < 0.05), but they were not significant when comparing NI to SI groups.

### 2.8. Protein Expression of Various Cytokines by M0 Macrophages Treated with TiO_2_

**Figure 7:**
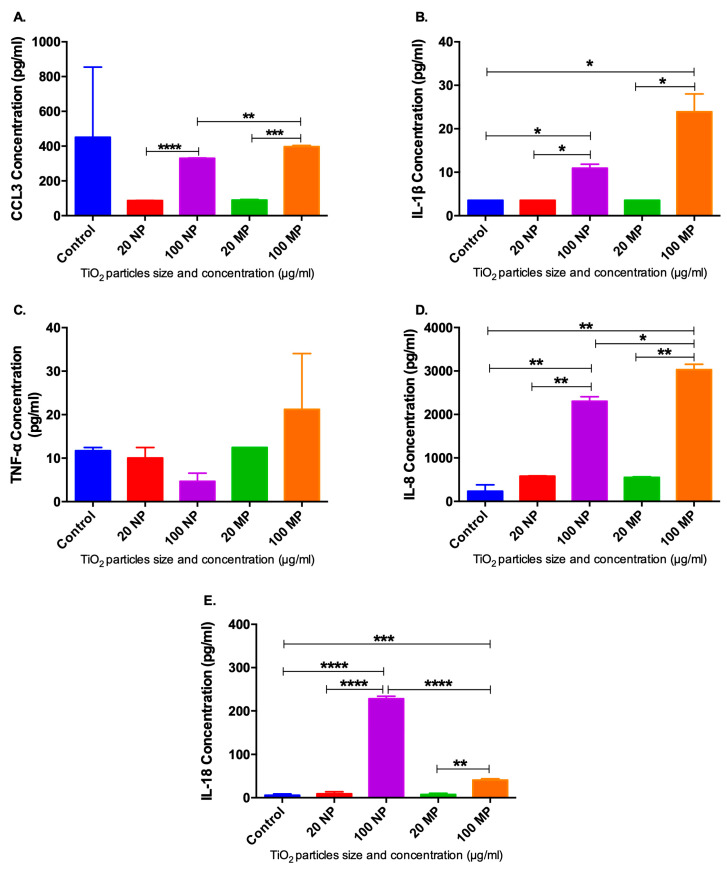
Protein level expression of cytokines by M0 macrophages treated with TiO_2_. (**A**) CCL3, (**B**) IL-1β, (**C**) TNF-α, (**D**) IL-8, and (**E**) IL-18 protein expression M0 macrophage after being treated for 24 h with 20 and 100 µg/mL of TiO_2_ NPs and MPs using Luminex/ELISA. The expression of cytokines is represented in terms of picogram /milliliter to non-treated M0 macrophages. Data are represented as mean ± SEM from two separate experiments. * *p* < 0.05, ** *p* < 0.01, *** *p* < 0.001, and **** *p* < 0.0001.

Various macrophage inflammatory-associated cytokines were investigated upon the treatment of M0 macrophages for 24 h with 20 and 100 µg/mL of TiO_2_ NPs and MPs. Interestingly, there was an increase in the release of IL-1β, IL-8 and IL-18 by M0 macrophages after being treated for 24 h with 100 µg/mL of TiO_2_ NPs (*p* < 0.05, *p* < 0.01 and *p* < 0.0001, respectively) and MPs (*p* < 0.05, *p* < 0.01 and *p* < 0.001, respectively) compared to untreated cells. Furthermore, there was a significant increase in the levels of IL-1β, CCL3, and IL-8 between the 20 and 100 µg/mL treatments of TiO_2_ NPs and MPs. However, TNF-α did not show any significant change upon different treatments of TiO_2_ (Figure 7).

### 2.9. Protein Interaction Network of the Significantly Altered Cytokines in the Peri-Implant Gingival Tissues with the Presence of TiO_2_

**Figure 8:**
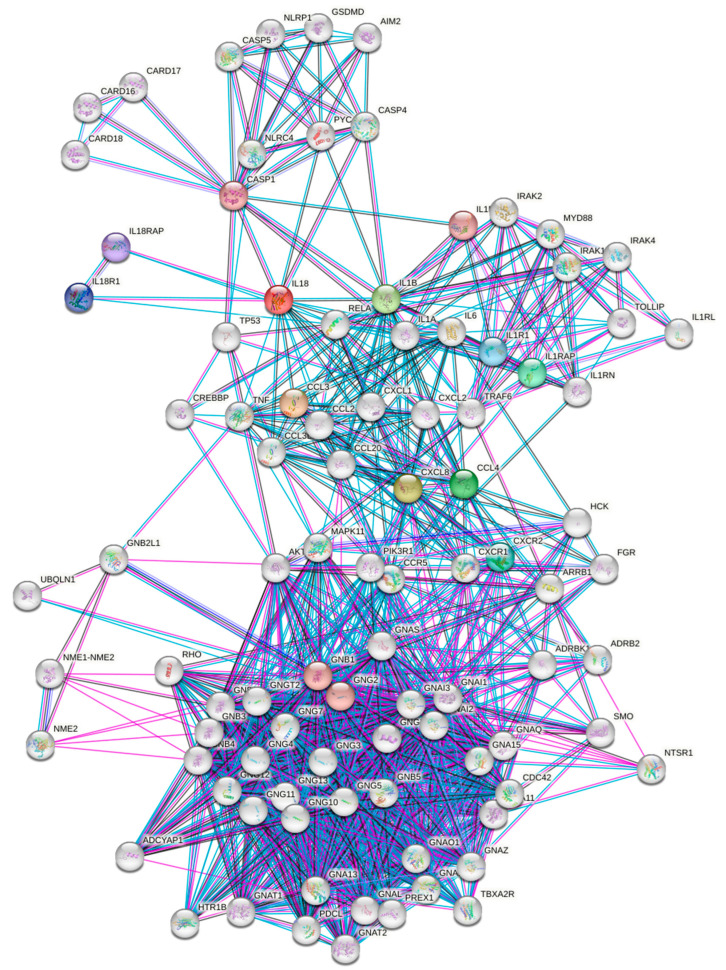
Protein–protein interaction network of altered cytokines in the peri-implant gingival tissues. The network nodes present proteins. Differently colored lines represent six types of indicators used in predicting the network associations; red line: the presence of fusion evidence; green line: neighborhood evidence; blue line: co-occurrence evidence; purple line: experimental evidence; light blue line: database evidence; black line: coexpression evidence.

We constructed protein interaction networks of the significantly altered cytokines in the peri-implant gingival tissues (CCL3, IL-1β, IL-8, and IL-18) which were validated in the M0 macrophages treated with TiO_2_. The interaction network analysis showed that all cytokines are functionally interconnected (Figure 8). Analysis of the protein interaction network showed the presence of several DEGs in FI compared to NI, including AKT1, GNA15, GNAI1, GNB2L1, GSDMD, NLRC4 and SMO that showed potential interactions and commonly affect each other’s expression and function. Thus, it can be supposed that the disruption of the cytokines CCL3, IL-1β, IL-8, and IL-18 plays an essential role in dental implant osseointegration.

## 3. Discussion

To the best of our knowledge, this is the first study using the transcriptome analysis to compare peri-implant gingival tissues from FI, SI and NI patient groups. In the present study, the distinct gene expression profiles in transcriptome analysis between tissue samples from FI, SI and NI could be considered an indication of the impact of titanium particles in the gene expression changes. Moreover, this may be correlated to the infiltration of inflammatory cells, which confirms the unique immune fraction in each subgroup resulting in the hierarchical clustering and heatmap analyses. Furthermore, the data extracted from the in silico flow cytometry analysis confirmed the profusion of macrophages in the peri-implant tissues with variations in the M1 with a 9.4% and 9.9% ratio and the M2 with a 22.3% and 18.7% ratio, respectively, in FI and SI tissues compared to NI samples. The percentage of M2 macrophages and T cells in the tissue from cases with FI was more than that in SI, but the percentage of M1 macrophages was more in SI than that in FI. This finding is in accordance with orthopedic studies that have reported an abundance of CD68 macrophages in periprosthetic orthopedic tissues containing wear metal debris [18] and an increased number of CD68 macrophages in an aseptically loosened orthopedic prosthesis. Yang et al., analyzed mice implanted with human periprosthetic tissues and reported CD68 cells in much higher quantities when titanium particles were present compared to those free of titanium particles [23]. This increase in CD68 cells was thought to be associated with an increased number of particles-engulfed monocytes at sites containing titanium particles and wear debris. Our findings showed that M1 and M2 macrophages were detected in peri-implant gingival tissues from patients with FI and SI, which followed other studies where the plasticity and polarization of macrophages have been reported in the context of peri-implantitis [24]. Further future studies, which consider the use of CD163 and CD86, will be undertaken in order to investigate the polarization of M2 and M1, respectively.

The functional clustering and pathway enrichment of the differentially expressed genes in FI vs. NI, or SI vs. NI, identified various molecular pathways and biological processes. However, the immune response pathways were the most frequent in both comparisons, suggesting the important roles of the infiltrating immune cells in the peri-implant tissues. For the DEGs in the FI group, we showed that B cells and T cells activation and proliferation processes were upregulated, although the cellular responses to chemical stress and response to alcohol were under-regulated in FI compared to NI. Indeed, for the DEGs in the SI group, different pathways related to the immune response, process and activation of immune cells were upregulated, including leukocyte migration, myeloid leukocyte activation, neutrophil and leukocyte degranulation, in addition to the regulation of cytokine production. These findings are consistent with previous results showing that in peri-implant tissues, the gene expression changes are mainly related to immune responses and defense responses [25]. Additionally, the GSEA further showed that gene sets related to M1/M2 macrophage polarization, immune responses, immune cell activation and proliferation and cytokine signaling were differentially over-represented between the different subgroups. Importantly, the leading-edge analyses revealed important differentially activated genes that are involved in multiple inflammations and immune responses related to biological processes or molecular pathways, including IL-1β, CDK3, IL-27 and CD86 in the FI vs. NI comparison, in addition to CXCL6, CXCL1, CCL7, CCL13 and IL18 in the SI vs. NI comparison. Previous transcriptomics studies profiling the differentially expressed genes in peri-implantitis have pointed out that the significantly altered pathways were related to extracellular matrix molecules [26], matrix-degrading enzymes [27] and inflammatory pathways including the RANKL/OPG pathway and the cyclooxygenase2 pathway [28]. 

On the other hand, the functional enrichment analysis of the DEGs common to FI and SI peri-implant gingival tissues showed that significant enrichment of transcripts is involved in response to stimulus, immune system defense, detoxification and signaling, indicating that in both cases of failed and successful implants, there is a release of the titanium which is considered as stress for the cells in the peri-implant area and therefore, it results in the triggering of the immune system, activating mainly M1 macrophage polarization, and maybe later, M2 polarization. In addition, it can thus be assumed that an imbalance of macrophage M1–M2 polarization is often associated with various inflammatory conditions, which leads to implant failure in some cases and success in others. This finding is in agreement with the Tilton et al. study, and further supports the idea that the exposure of THP-1 monocytes to TiO_2_ particles may induce the biological processes and differential regulation of inflammation involved in neutrophil activation and immune responses such as phagocytosis [29]. These results are in good agreement with our earlier study which showed that the phagocytosis of TiO_2_ particles by macrophages is size- and dose-dependent [30].

In the present study, the images from H&E slides confirmed the presence of titanium particles and infiltration of inflammatory cells in the peri-implant gingival tissues collected from FI or SI patients. The visual examination of the images showed that titanium particles were present in approximately 85% of the tissue samples, signifying that titanium particles often dislodge from the implant surface. However, the expression of inflammatory cells in the FI samples was higher than that in the SI samples. This finding refers to the role of titanium particles to increase inflammation in the peri-implant gingival tissues. As proposed forward by Tawse-Smith et al. [7], the titanium particles were found highly abundant in all peri-implantitis tissue when compared with other test and control areas using SEM-EDS analysis. We showed that the inflammatory cells infiltrating the peri-implantitis tissues were primarily chronic, which is a common finding for peri-implantitis tissues. This is in agreement with a study by Dapunt et al. who found that the release of metal ions from the prosthesis can induce an acute phase of the chronic inflammatory disease with infiltrates of monocytes, T cells, and osteoclasts [31]. Nevertheless, the influence of microbial factors will never be neglected in the development of peri-implantitis, though, the main focus of the current study was investigating the infiltration of titanium particles and inflammatory cells into peri-implant gingival tissues and the consequences of this fact. Hence, additional assessments in the gingival tissue to identify the content of titanium particles will be warranted for future research, which will pave the way to a better understanding of the association between the content of titanium particles in the gingival tissues and chronic inflammation, peri-implantitis and failure of osseointegration. 

The images from the IHC analysis showed positive staining of CD68 and CD3 in the presence of titanium particles, and the expression of the positive staining in FI cases was more than that in SI cases. This could be an indication of the increase in the infiltration of macrophages and lymphocytes into the gingival tissues around FI compared to the SI tissues, which may refer to the role of titanium particles in macrophage polarization. This finding is in agreement with the clinical study by Rao et al. who stated that the failure of orthopedic implants was related to the presence of excessive M1-polarized macrophages around titanium joints [32]. Additionally, Galarraga Vinueza et al. showed in the IHC study that there was an increase in the M1/M2 macrophages ratio in the peri-implant gingival tissues from patients with peri-implantitis [33]. Consequently, this may be considered an important factor related to the failure of the implant and could lead to absorption of the surrounding bone and loosening of the implants.

In the clinical scenario, the whole cells in the peri-implant gingival tissues are usually exposed to titanium wear particles. However, in the in vitro experiments used in the validation study, we targeted only macrophages as the main immune cell included in the response to foreign body reactions. Worthy of consideration is that IHC analysis confirmed the infiltration of macrophages into the peri-implant gingival tissues collected from FI and SI with the presence of titanium particles and the identified related genes by GSEA in both comparisons. Thus, the functional validation was crucial to confirm the impact of titanium particles in the polarization of M1 macrophages which are responsible for the expression of inflammatory cytokines such as Il-18, Il-1β, CCL3, TNF-α, and IL-8.

In the present study, the size of the titanium particles was not possible to recognize in the IHC and H&E images due to the potential modification caused by the microtome cutting technique, which limits the thickness of the samples. Therefore, to replicate the clinical scenarios, we used different concentrations of nano- and micro-sized TiO_2_ particles to treat THP-1 monocyte-derived M0 macrophages, thereby validating the findings obtained from the IHC analysis. The results at the protein level confirmed that TiO_2_ particles induce the secretion of IL-1β by macrophages. This finding was in agreement with previous evidence demonstrating that IL-1 serves as a pro-inflammatory mediator expressed by macrophages [34]. Additionally, our findings showed an upregulation in the expression of macrophage markers in the tissue samples from the FI compared to SI. It is worth noting that IL-1β was associated with the direct differentiation of pre-osteoclasts into osteoclasts, which potentially influenced the failure of dental implant osseointegration [35]. Furthermore, the activation of NF-κB involves numerous molecules, including IL-1β and TNF-α. Excitingly, the IL-1β promoter contains a functional NF-κB binding site, and this indicates that IL-1 positively autoregulates its synthesis as it acts as a potential inducer of NF-κB binding activity. Accordingly, the IL-1β gene may be considered an important additional member of the cytokine gene family that is regulated in part by the NF- NF-κB/rel family of transcription factors [36].

The immune response to titanium particles is a complex process influenced by the interaction between numerous immune cells. These cells are exposed to the particles and produce different cytokines, which in turn induce the activation of pro-inflammatory macrophages. In our present study, there was upregulation of these pro-inflammatory cytokines by macrophages, indicating the potential link between the release of titanium particles and the failure of dental implants. This finding is in line with previous research demonstrating that IL-1β and inflammatory cytokines impact the activation of osteoclast and are associated with the downregulation of type 1 collagen. Consequently, the IL-1β cytokine is implicated in the resorption of bone and peri-implantitis [37]. TNF-α plays a crucial role in organizing the inflammatory response and the infiltration of immune cells. While no significant alterations were observed in the protein level of TNF-α following various treatments of TiO_2_, it is broadly recognized that TNF-α, in conjunction with IL-1β provokes downstream signaling pathways, such as mitogen-activated protein kinases (MAPKs) and the transcription factor NF-κB. These pathways eventually lead to the release of additional pro-inflammatory chemokines and cytokines [38].

CCL3 plays a crucial role in the recruitment and activation of macrophages, especially at sites of infection or inflammation. Its release stimulates other innate immune cells such as neutrophils, lymphocytes and dendritic cells. Moreover, CCL3 has been identified as a regulator of acute and chronic inflammatory responses, and it acts as a potent activator of osteoclasts, leading to bone resorption [39]. As previously mentioned, TNF-α and IL-1β were reported to control the transcription and expression of CCL3 through the MAPK and NF-κB pathways [21]. Our study observed an upregulation of IL-8 expression in response to the treatment of M0 macrophages with TiO_2_, which aligns with previous findings demonstrating upregulation of IL-8, IL-1β and TNF-α production macrophages [34]. These findings are in agreement with previous studies indicating that excessive IL-8 production can affect the inflammatory response, leading to delayed tissue healing and impairment in endothelial tube growth [40]. The expression of IL-18 at the protein level was found to be increased following the treatment of M0 macrophages with TiO_2_ particles. Importantly, IL-18 is involved in the priming of other immune cells such as NKT cells, leading to the expression of TNF-α, which is essential for the differentiation of osteoclasts [41].

The collection of gingival tissue samples from SI is not feasible since the explantation of well-osseointegrated implants in humans to get gingival tissue samples is a rarity to happen and is an exception [42]. Hence, the gingival tissue that is covering the SI was resected during the routine uncovering procedure stage to place the healing abutment (3–5 months after implant placement) and used as SI gingival tissue samples. Consequently, the most common reason for titanium particles’ release into gingival tissue around SI could be the friction contact between the implant and alveolar bone during implant placement [43]. Those implants were considered as SI as there was successful osseointegration and no signs of peri-implantitis. The peri-implant gingival tissue samples from the FI were collected 1–2 years after the connection of the crown to the implant body; thus, the micro-movement/friction between the implant and its crown might be the reason for the development of a micro-gap at an implant-crown interface that could be considered as the source for titanium particles [44,45]. Worth noting, all implants included in the study were of the same model surface characteristics. In the present study, iatrogenic contamination of metal elements into the peri-implant gingival tissues was not probable as meticulous care was taken during the harvesting of the tissue samples to prevent the accidental release of titanium. This was in accordance with previous studies where the tissue samples were collected prior to the removal of the implants to prevent contamination with metal from corrosion happening at the time of implant removal [46].

**Figure 9:**
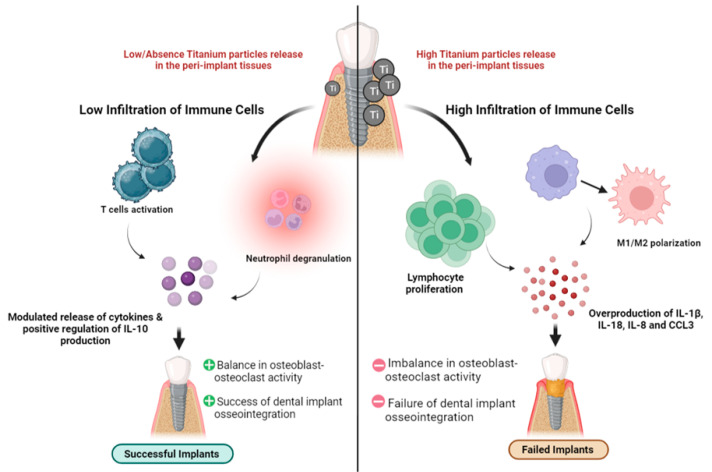
Summary of the study findings: schematic representation of the infiltration of titanium particles and immune cells into the peri-implant gingival tissues.

The findings of the present study will facilitate our understanding of immune cell population-specific molecular events deriving the peri-implant inflammation in response to titanium particles. Furthermore, the activated inflammatory mediators may be the key to the imbalance in osteoblast–osteoclast activity, failure of osseointegration and dental implant treatment which paves the way for discovering potential molecular mechanisms underlying both diseases, diagnostic biomarkers and novel therapeutic perspectives (Figure 9). Despite that, this study has limitations. For the in vitro validation, THP-1 monocyte-derived macrophage cell lines were used instead of those derived from the patients due to the limited source of patient samples. Moreover, the small sample size was a limitation in this study, but we were able to overcome this limitation by using different bioinformatics tools to identify significant differentially expressed genes and confirm the differentially activated molecular pathways and biological processes in each comparison of FI, SI and NI groups.

## 4. Materials and Methods

**Figure 10:**
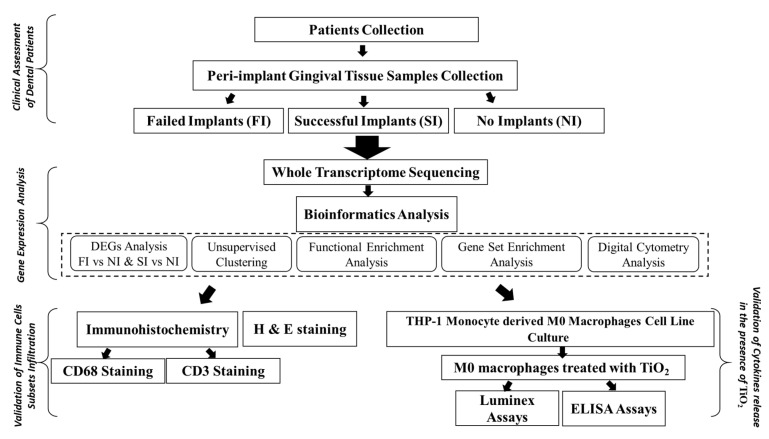
General flowchart of the research methodology.

The study was approved by the Research Ethics Committee at the University of Sharjah (REC-19-10-30-01). All patients who volunteered as subjects in this study were informed about the study conditions, asked for their participation, and given written consent for the surgical procedure. Subjects were divided into three groups: Failed Implants (FI) group, Successful Implants (SI) group and No Implants (NI) group. The study methodology is detailed in (Figure 10). 

### 4.1. Patients’ Selection Criteria

The inclusion criteria were (i) patients with an implant-supported fixed or removable prosthesis suffering from peri-implantitis and indicated for explantation (removal of the implant) [2]; these were considered in the FI group. Peri-implantitis was defined as the existence of signs of peri-implantitis represented by clinical and radiographical findings, such as bleeding on probing, probing depth beyond 5 mm and radiographic bone loss of more than 3 mm around the dental implant with or without cortical bone perforations, that is related to the mobility and non-restorability of the implant [2]. The inclusion criteria also consisted of (ii) patients with dental implants indicated for second-stage surgery and healing-abutment fixation, without any signs of peri-implantitis; these were considered in the SI group. Lastly, the inclusion criteria also included (iii) patients with partially impacted wisdom teeth without pericoronitis, who were considered in the NI group. The exclusion criteria were patients younger than 18 years and above 60 years, patients with systemic disease, smoking and alcohol consumption, and patients who had previous periodontal therapy of the dental implant (to avoid iatrogenic causes of titanium release into the peri-implant tissues). Moreover, patients with periodontal disease of the teeth adjacent to the implants were excluded from the study.

### 4.2. Collection of Gingival Tissue Samples

The peri-implant gingival tissue samples were collected from patients who attended the University Dental Hospital Sharjah for dental implant treatment or extraction of wisdom teeth. All patients went through clinical examination to be sure that the teeth adjacent to the implants are without periodontitis, the gingival index is 0, the plaque index is 0, and the probing depth is about 1–2 mm; besides, the dental history showed no history of periodontitis. A total of 20 patients were selected based on the inclusion and exclusion criteria of the study. The age and gender distribution of the patients included in the study are summarized in the Supplementary Table S1. Peri-implant gingival tissue samples were collected from areas adjacent to the implants and contained almost a similar content of both epithelium and connective tissue. The peri-coronal gingival operculum tissues from impacted wisdom teeth sites were collected as well. 

The gingival tissue samples of the FI group were taken from eleven patients with peri-implantitis. The implant was indicated for explantation (1–2 years after implant loading). Under local anesthesia, a circular incision with releasing incisions mesial and distal of the implant was performed in the gingival tissues using a 15C scalpel (Sigma Aldrich, St. Louis, MO, USA). Then, 4 mm from the crest of the gingival tissue around the FI was excised, followed by the surgical removal of the dental implant. The gingival tissue samples of the SI group were taken from six patients who attended the clinic for second-stage surgery to uncover the implants and placement of healing abutments (3–5 months after the implants’ placement). Under local anesthesia, the regular protocol for uncovering dental implants was followed using a hemostatic clamp and 15C scalpel, and 4 mm of the gingival tissue that was excised to uncover the implant was used as an SI tissue sample. The gingival tissue samples of the NI group were taken from three patients who attended the implant clinic for the surgical extraction of wisdom teeth. The regular surgical protocol for the surgical extraction of a wisdom tooth was performed, followed by the excision of 4 mm of healthy peri-coronal gingival tissue at the gingival crest of the extracted tooth. 

### 4.3. Processing of Gingival Tissue Samples and Paraffin-Embedded Blocks’ Preparation

Gingival tissue samples from the three groups were fixed in 10% neutral buffered formalin (Thermo Scientific, Waltham, MA, USA). After 2 days of fixation, tissues were processed by an Excelsior AS tissue processor (Thermo Scientific, Waltham, MA, USA) using a routine overnight protocol and embedded as paraffin blocks (TEC 2900, Pantigliate MI, Italy). Plain tissue slides (Medix, Newbury Park, CA, USA) were prepared in 3–5 μm sections with the HM 355S Automatic Microtome (Thermo Scientific, Waltham, MA, USA), deparaffinized and then rehydrated with graded ethanol (Sigma-Aldrich, St. Louis, MO, USA).

### 4.4. Whole Transcriptome Analysis

#### 4.4.1. RNA Sequencing

The paraffin blocks of the FI, SI and NI samples were sectioned into slides at 5μm thickness using the HM 355S Automatic Microtome (Thermo Scientific). Then, the tissues were macrodissected from the slides and collected in Eppendorf tubes for RNA extraction as previously described [47]. The RNA was extracted using the RNA RecoverAll kit, followed by the addition of Turbo DNAse and further cleaned and concentrated using the Zymo RNA mini-Kit according to the manufacturer’s instructions (Zymo Research, Irvine, CA, USA). Purified RNA was used for whole transcriptome profiling analysis using targeted RNA-Seq with the Ion AmpliSeq Whole Transcriptome human gene expression kit (Thermo Fisher Scientific), which is designed to target over 21,000 specific human RNA transcripts using a high-throughput multiplexed method as previously described [48]. RNA sequencing was performed using an Ion 540 Chip on an Ion S5 XL Semiconductor sequencer (Life Technologies, Carlsbad, CA, USA).

#### 4.4.2. RNA-Seq Data Analysis

The RNA-seq data were analyzed using the Ion Torrent Software Suite version 5.4. The Torrent Mapping Alignment Program (TMAP) was used to align the raw sequencing reads to the GRCh37/hg19 Human reference genome. The raw read counts of the targeted genes were normalized using the Fragments Per Kilobase Million (FPKM) normalization method. The number of expressed transcripts was evaluated in the subsequent quality control step. A minimum of 10 million total reads per sample was recommended for further analysis. Next, the DESeq2 R/Bioconductor package (https://bioconductor.org/packages/release/bioc/html/DESeq2.html) (accessed on 21 September 2021) was used to identify the differentially expressed genes (DEGs) in each comparison of patients with FI and SI against patients with NI. DESeq2, the negative binomial generalized linear model fitting was applied and the Wald test was performed to analyze the abundance data. Read count normalization was carried out using the regularized logarithm (rlog) method provided in DESeq2. Genes with low read counts were filtered out; only genes with more than ten normalized read counts were considered for further analysis. DEGs were selected with Fold change (FC) > 2 at a significance level of the adjusted *p*-value (FDR) < 0.05.

#### 4.4.3. Clustering and Functional Enrichment Analysis of the Differentially Expressed Genes

In order to confirm the classification of the studied patients, unsupervised clustering into distinct subclusters was performed based on the expression patterns of the DEGs lists between patients with FI and SI against patients with NI independent of their clinic-pathological features or intrinsic molecular subtypes. This was carried out using Euclidean distance measure and Ward linkage [49]. Moreover, the obtained upregulated and downregulated gene lists between patients with FI and SI against patients with NI were annotated and analyzed using the Metascape tool [50]. The latter is relying largely on well-annotated pathways derived from Gene Ontology (GO), Reactome and KEGG databases. Indeed, the DEGs of the two comparisons of FI and SI against NI were intersected to identify the commonly differentially regulated genes between the failed and successful implants using InteractiVenn [51]. The common genes were further analyzed for pathways annotation.

#### 4.4.4. Gene Set Enrichment Analysis

The significant DEGs lists obtained were further analyzed to identify the enriched pathways and the related genes in the peri-implant inflammation in response to titanium particles in comparison to normal tissues using Gene Set Enrichment Analysis (GSEA). A GSEA search was performed in large sets of cellular pathways and biological processes obtained across Immunome, Immunologic signature gene sets C7 and ontology gene sets C5 from the GSEA database (https://www.gsea-msigdb.org) (accessed on 30 November 2021). The significantly enriched pathways with *p*-value (*p*) < 0.05 were further explored to identify the top relevant genes in the respective sets.

#### 4.4.5. In Silico Prediction of the Immune Cells’ Subsets in the Peri-Implant Gingival Tissues

In silico CIBERSORT, a digital cytometry that can identify cell type composition and expression from bulk tissues, was carried out on the DEGs gene lists to identify the composition of immune cells in the peri-implant gingival tissues of each subgroup of FI and SI patients compared to the NI group. As references, we used gene lists for the immune cells including naïve CD8 T cells, naïve CD4 T cells, alternatively activated macrophages, classically activated macrophages, regulatory T cells, T helper cells, natural killer cells, and dendritic cells [52].

### 4.5. Histological and Cytological Staining

To validate the transcriptome findings concerning the infiltration of titanium particles and immune cells represented by macrophages and lymphocytes, immunohistochemistry (IHC) and Hematoxylin–Eosin (H&E) analyses were performed. The peri-implant gingival tissue samples from the same selected FI, SI and NI patients were used in the IHC and H&E assessment. The slides were subjected to antigen retrieval of CD3 and CD68 markers (Table 4) by steaming them in an appropriate retrieval buffer for 20 min, followed by blocking with 5% bovine serum albumin (BSA) for 30 min. Primary antibody incubation was performed by overnight incubation at 4 °C with desired antibody dilution in 1% BSA solution and followed by 1 h incubation with the horseradish peroxidase (HPR)-labeled suitable secondary antibody dilution in 1% BSA solution. The color was developed with 3,3′-Diaminobenzidine (DAB) (Abcam, Cambridge, UK) and counterstained with Shandon Harris Hematoxylin (Thermo Scientific). For the two antibodies, the EDTA buffer with a pH of 8.0 for heat-induced antigen retrieval during immunohistochemistry (IHC) and the Ventana autostainer (Ventana-Benchmark XT, Roche Inc., Branchburg, NJ, USA) were used. Counterstaining was performed using Gill’s hematoxylin (GHS1128, Sigma-Aldrich, USA) and slides were rinsed in a container with cold running water. To confirm the specificity of the antibody sections with human samples, tonsil tissues were used as appropriate positive controls. The images were captured with an Olympus DP74 microscope digital camera attached to a BX43 microscope (Olympus Life Sciences, Tokyo, Japan), at various magnification powers. H&E and IHC staining was validated by two experienced pathologists (IT and RAH). 

### 4.6. Validation of Cytokines’ Release in the Presence of TiO_2_

Given the results of previous steps, certain cytokines of interest were explored in THP-1 monocyte-derived M0 macrophage cell lines treated with TiO_2_ particles.

#### 4.6.1. THP-1 Monocyte-Derived M0 Macrophages Cell Line Culture

First, to generate THP-1 monocyte-derived M0 macrophages cell lines, THP-1 monocytes were obtained from CLS cell line services (CLS cell line services GmbH, Eppelheim, Germany) and maintained in an RPMI-1640 medium supplemented with 10% fetal bovine serum (FBS), 100 U/mL of penicillin and 100 µg/mL of streptomycin. The cells were maintained in a humidified environment with 5% CO_2_ and 37 °C. THP-1 monocyte cells were further converted into M0 macrophages using 100 ng/mL of phorbol 12-myristate 13-acetate (PMA) (Sigma-Aldrich, USA) for 48 h according to the protocol previously described [44]. Following exposure to PMA, the cells were maintained in a PMA-free medium for 48–72 h. The flow cytometry analysis using CD14 was used to confirm the differentiation of the THP-1 monocyte into M0 macrophages.

#### 4.6.2. Titanium Dioxide Solutions’ Preparation

Titanium dioxide (TiO_2_) powders with a primary particle size of <100 nm nanoparticles (NPs) and <5 µm microparticles (MPs) in diameter were used (Sigma-Aldrich, USA). For stock preparation, 10 mg of TiO_2_ NPs and MPs were weighed in separate 15 mL tubes and dispersed in 10 mL of Milli-Q water using a sonicator equipped with a 3.2 mm diameter microtip (Qsonica sonicators, Qsonica, Newtown, CT, USA) operated at 40% in pulse mode (50 s on /50 s off). The total sonication cycle lasted up to 10 min. For further analysis, TiO_2_ particles were suspended in a complete RPMI-1640 medium to a final concentration of 1 mg/mL (stock suspension). The hydrodynamic diameter, polydispersity index (PDI), and effective charge of TiO_2_ NPs and MPs were measured with a Malvern Zetasizer Nano-ZS system (Malvern Instruments, Malvern, Worcestershire, UK). All measurements were performed in complete cell culture media at 25 °C using a particle concentration of 20 and 100 μg/mL for NPs and MPs. The TiO_2_ particle size was also analyzed using SEM (VEGA3 XM-TESCAN, Brno-Kohoutovice, Czech Republic). The prepared TiO_2_ NPs and MPs solutions were dropped onto Aluminum stubs and air-dried and later coated with Gold-Palladium and the particle size and morphology were analyzed under SEM.

#### 4.6.3. Validation of Cytokines’ Protein Expression

We further assessed the alterations in the concentration of cytokines of interest *CCL3, TNF-α, IL-1β, IL-18* and *IL-8* by the THP-1 monocyte-derived M0 macrophage after preferred treatments in the culture supernatants for 24 h with 20 and 100 μg/mL of TiO_2_ NPs and MPs. CCL3, TNF-α, IL-1β and IL-8 cytokine levels were investigated using a human cytokine magnetic Luminex panel (R&D systems, Minneapolis, MN, USA) and the Bioplex-200 detection system (BioRad Laboratories, Hercules, CA, USA); IL-18 was assessed using a human IL-18 ELISA kit (Abcam, Cambridge, UK). The experiments’ details are described in the Supplementary Material.

### 4.7. Protein–Protein Interaction (PPI) Network Analysis

The protein–protein interaction network analysis permits the visualization of the functional relationships between proteins [53]. To identify potential interactions between the significantly altered cytokines in the peri-implant gingival tissues, the STRING tool was employed to construct the PPI network. Several interaction sources including experiments, databases, physical interactions and co-expression at a minimal confidence interaction score of 0.4 were applied to construct the PPI network between the input proteins and their neighbors at ten more layers.

### 4.8. Statistical Analysis

The Kruskal–Wallis test was used for IHC analysis between the three groups of FI, SI and NI. The post hoc Tukey test and one-way ANOVA were used for analyzing ELISA. For the Luminex assay, the unpaired Student’s *t*-test was used to compare each of the two groups. *p* < 0.05 was statistically significant.

## 5. Conclusions

This study aimed to investigate the immune microenvironment that is involved in the peri-implant inflammation induced by titanium particles. Accordingly, the peri-implant gingival tissue samples were collected from patients with failed implants, successful implants, and no implants. The analysis of whole transcriptome data found a significant enrichment of transcripts related to immune response signaling pathways, particularly T cell-mediated immunity and M1/M2 macrophage polarization. Moreover, histological staining (H&E and IHC) confirmed the presence of lymphocytes, macrophages, and titanium particles in the peri-implant gingival tissues of both failed and successful implants. Furthermore, the functional validation showed a remarkable increase in the expression levels of IL-18, IL-1β, IL-8 and CCL3 by THP-1 monocyte-derived macrophages when exposed to different concentrations of titanium dioxide particles. These findings will contribute to highlighting the role of titanium particles in the development of peri-implant inflammation and failure of dental implant treatment. Thus, further research is warranted to investigate the early detection of titanium particle infiltration into peri-implant gingival tissues and understand the changes in immune cell composition in the affected peri-implant environment.

## Figures and Tables

**Table 1:** Functional and pathway analyses of the differentially expressed genes in gingival tissues from SI and FI compared to NI.

Group	Expression	Description	Category	Gene Count	*p*-Value
Successful Implants (SI)	Up regulated	Detection of chemical stimulus	Biological Processes: GO:0009593	55	1.12 × 10^−7^
		Leukocyte migration	Biological Processes: GO:0050900	37	5.77 × 10^−5^
		Myeloid leukocyte activation	Biological Processes: GO:0002274	26	6.33 × 10^−5^
		Neutrophil degranulation	Reactome Gene Sets: R-HSA-6798695	44	8.49 × 10^−5^
		Regulation of cytokine production	Biological Processes: GO:0001817	64	1.44 × 10^−4^
		Cellular defense response	Biological Processes: GO:0006968	10	2.3 × 10^−4^
		Leukocyte degranulation	Biological Processes: GO:0043299	12	3.23 × 10^−4^
		Positive regulation of interleukin-10 production	Biological Processes: GO:0032733	8	8.28 × 10^−4^
		Apoptotic cell clearance	Biological Processes: GO:0043277	9	9.91 × 10^−4^
	Downregulated	Cellular responses to stimuli	Reactome Gene Sets: R-HSA-8953897	422	3.88 × 10^−54^
		Programmed cell death	Reactome Gene Sets: R-HSA-5357801	50	2.83 × 10^−15^
Failed Implants (FI)	Upregulated	Cellular response to cadmium ion	Biological Processes: GO:0071276	10	1.9 × 10^−3^
		Lymphocyte proliferation	Biological Processes: GO:0002376	12	3.4 × 10^−4^
		B-Cell activation	Biological Processes: GO:0042113	21	1.92 × 10^−5^
		T-Cell activation involved in immune response	Biological Processes: GO:0002286	9	4.69 × 10^−3^
	Downregulated	Cellular response to chemical stress	Reactome Gene Sets: R-HSA-9711123	47	4.7 × 10^−6^
		Response to alcohol	Biological Processes: GO:0097305	24	1.9 × 10^−6^

**Table 2:** Significantly enriched pathways are differentially activated between FI and SI compared to NI.

Comparison	Gene Set	Source	Size	ES	NES	NOM *p*
FI vs. NI	M1 vs. M2	GSE5099	23	0.62	1.64	<0.001
	CD4 EFF MEM VS PBMC DN	GSE11057	21	0.52	1.49	<0.05
	LYMPHOCYTE PROLIFERATION	GO:0008283	50	0.40	1.52	<0.05
	CSF1 IFNG VS CSF1 IFNG PAM3CYS IN MAC UP	GSE11864	15	0.53	1.54	<0.05
	UNTREATED VS CSF1 IFNG PAM3CYS IN MAC DN	GSE11864	16	0.53	1.82	<0.001
	TFH VS TH17 CD4 TCELL DN	GSE11924	15	0.62	1.60	<0.001
	IGD POS BLOOD VS NAIVE TONSIL BCELL DN	GSE12845	17	0.63	1.42	<0.001
	IMM VS MATURE NKCELL DN	GSE13229	16	0.60	1.66	<0.05
SI vs. NI	NAIVE VS MEMORY CD8 TCELL DN	GSE10239	47	0.49	1.58	<0.001
	CD4 TCELL VS LUPUS CD4 TCELL DN	GSE10325	35	0.56	1.59	<0.001
	NAIVE VS CENT MEMORY CD4 TCELL UP	GSE11057	30	0.54	1.69	<0.001
	NAIVE VS EFF MEMORY CD4 TCELL UP	GSE11057	41	0.54	1.71	<0.001
	NAIVE VS DAY8 EFF CD8 TCELL UP	GSE1000001	41	0.59	1.38	<0.001
	RESPONSE TO CHEMICAL STIMULUS	GO:0042221	63	0.44	1.64	<0.001

ES: Enrichment Score; NES: Normalized ES; NOM: nominal.

**Table 3:** The relative percentage of the immune cells’ subsets distribution in FI and SI. The data were extracted from the *CIBERSORT* in silico flow cytometry analysis.

Immune Cells	Successful Implants SI (%)	Failed Implants FI (%)
Memory CD8 T-Cells	6.8	6
CD8 T-Cells	13.4	0
Regulatory T cells (Tregs)	3.15	16.4
M1 macrophages	9.9	9.4
M2 macrophages	18.7	22.3
CD4 T-Cells	19.6	45.9

**Table 4:** Types of markers and dilutions of antibodies used in immunohistochemistry analysis of peri-implant gingival tissue samples.

Target	Retrieval Condition	Primary Antibody Cat. No.	Dilution Used of Primary Antibody	Secondary Antibody Cat. No.	Dilution Used of Secondary Antibody
CD3	PH-9, Tris-EDTA buffer	PA5-79720	1:3000	ab 205718	1:4000
CD68	PH-6 Sodium citrate	EPR-20545	1:50	ab 201795	1:500

## Data Availability

All relevant data are available in the paper and the Supplementary Materials. The raw RNA-seq data are deposited to a public repository and can be accessed from https://doi.org/10.6084/m9.figshare.21360498 (accessed on 19 December 2022).

## Supplementary Materials

The following supporting information can be downloaded at: https://www.mdpi.com/article/10.3390/ijms241411644/s1.
