# Supplementary material for: Titanium Particles Modulate Lymphocyte and Macrophage Polarization in Peri-Implant Gingival Tissues
Source: Int J Mol Sci. 2023 Jul 19;24(14):11644. doi: 10.3390/ijms241411644 (PMC10381089; doi:10.3390/ijms241411644)
Supplement: Supplementary file 1 [file ijms-24-11644-s001.zip › ijms-2485220-supplementary.pdf]

Supplementary Table S1

Age and gender distribution of the patients included in the study.

Group Name	Age mean (SD)	Gender Female	Male
**Failed Implants, FI**	59.91 (6.7)	6 (54.6%)	5 (45.4%)
**Successful Implants, SI**	41.17 (8.5)	2 (33.3%)	4 (66.7%)
**No Implants, NI**	31 (2)	2 (66.7%)	1 (33.3%)

Supplementary Material

Titanium dioxide solutions preparation

Titanium dioxide (TiO_2) powders with a primary particle size of <100 nm nanoparticles (NPs) and <5 µm microparticles (MPs) in diameter were used (Sigma-Aldrich, USA). For stock preparation, 10 mg of TiO_2 NPs and MPs were weighed in separate 15 mL tubes and dispersed in 10 mL of Milli-Q water using a sonicator equipped with a 3.2-mm diameter microtip (Qsonica sonicators, USA) operated at 40% in pulse mode (50 sec on /50 sec off). The total sonication cycle lasted up to 10 min. For further analysis, TiO_2 particles were suspended in a complete RPMI-1640 medium to a final concentration of 1 mg/mL (stock suspension). The hydrodynamic diameter, polydispersity index (PDI), and effective charge of TiO_2 NPs and MPs were measured with a Malvern Zetasizer Nano-ZS system (Malvern Instruments, UK). All measurements were performed in complete cell culture media at 25°C using a particle concentration of 10 µg/mL for NPs and 50 µg/mL for MPs.

Titanium dioxide particles characterization

The TiO_2 particle size was also analyzed using SEM (VEGA3 XM-TESCAN, Czech Republic). The prepared TiO_2 NPs and MPs solutions were dropped onto Aluminum stubs and air dried and later coated with Gold-Palladium and the particle size and morphology were analyzed under SEM.

Quantitative real-time polymerase chain reaction (qPCR)

To better understand the expression variations of the selected genes (IL-1β, IL18) in M0 macrophages treated with TiO_2, we proceeded as follows: THP-1-derived monocytes were seeded at 200,000 cells/well in a 6-well plate and differentiated into M0 macrophages as previously described. M0 cells were treated with two different concentrations of TiO_2 NPs and MPs (20 or 100 µg/mL) for 24 h. At definite time intervals, total RNA was extracted from cells using RNA isolation with the RNeasy Mini Kit (Qiagen, Hilden, Germany), according to the manufacturer's instruction. The quantity of isolated RNA was measured in a nanodrop spectrophotometer system (ND-1000, USA). The total RNA thus isolated was transcribed to cDNA using QuantiTect Reverse Transcription Kit (Qiagen, Hilden, Germany). The quantitative real-time polymerase chain reaction (qPCR) was carried out using 5x HOT FIREPol® EvaGreen® qPCR Mix Plus (Solis BioDyne, Estonia) in triplicates in a QuantStudio™ 3 Real-Time Applied biosystems PCR System (Thermo Fisher Scientific, USA). Briefly, the reaction volume (20µL) included 4 µl PCR master mix, 0.8µL each of forward and reverse primers (400 nM) and 2 µL of cDNA. The PCR cycling

conditions included an initial activation at 95°C for 12 min followed by 40 cycles of denaturation at 95°C for 15 sec, annealing at 59°C for 20 sec and an extension temperature at 72°C for 20 sec. Glyceraldehyde 3-phosphate dehydrogenase (GAPDH) (Abcam, Cambridge, UK) was used as the internal control. The expression levels of target genes (IL-1β, IL-18) were calculated by normalizing the Ct values with that of the reference gene and the fold change was expressed using the formula 2-ΔΔCt.

Luminex assays

The expression of IL-1β at the protein level was also investigated using the Luminex human multi-cytokine detection system. Frozen supernatants from human THP-1 monocyte-derived M0 macrophage cultures treated with 20 and 100 µg/mL TiO2 NPs and MPs were thawed rapidly at 37°C, mixed by vortexing, and spun at 10,000 x g for 5 min to remove any solid particles. Aliquots of 50µl were combined with coated beads. Commercial kits were run in individual plates, with their buffers and standards, following the directions of the manufacturer. Incubations and washes were performed in 1.2 µm filter membrane 96-well microtiter plates (MABVN1250, Millipore Corp., Billerica, MA). After the final wash, beads in the 96-well microtiter plate were resuspended in 125 µl Luminex sheath fluid and loaded into the magnetic Luminex instrument (R&D Systems, Inc., USA). An acquisition gate was set between 8,000 and 13,500 for the doublet discriminator, sample volume was 75 µl, and 100 events/regions were acquired. Raw data (mean fluorescence intensity) from all the bead combinations tested were analyzed with the Master-Plex QT quantification software (MiraiBio Inc., Alameda, CA) to obtain concentration values.

ELISA assays

ELISA measured the alterations in the secretion of IL-18 by the THP-1 monocyte-derived M0 macrophage after preferred treatments in the culture supernatants. Human IL-18 ELISA kits were purchased from Abcam (Abcam, UK), and ELISA was performed according to the manufacturer's instructions. Briefly, the culture supernatant was added to anti- IL-18 antibody-coated wells along with an antibody cocktail solution. After 1h of incubation at 37°C, the wells were washed three times with 1X wash buffer PT (Abcam, Cambridge, UK). Following this, 100 µl of tetramethyl benzidine (TMB) development solution (Sigma-Aldrich, Germany) was added and the plate was incubated with gentle shaking for 10 min in the dark. Then, the wells were added with 100µl stop solution and the relative absorbance was quantified using a microplate reader (Synergy HTX Multi-Mode Reader, Biotek Instruments, USA) at 450 nm. The concentration of IL-18 in the culture supernatant was calculated from a concurrently plotted IL-18 standard curve.
